# A Serverless Tool for Platform Agnostic Computational Experiment Management

**DOI:** 10.3389/fninf.2019.00012

**Published:** 2019-03-05

**Authors:** Gregory Kiar, Shawn T. Brown, Tristan Glatard, Alan C. Evans

**Affiliations:** ^1^Montreal Neurological Institute, McGill University, Montreal, QC, Canada; ^2^Department of Biomedical Engineering, McGill University, Montreal, QC, Canada; ^3^Department of Computer Science, Concordia University, Montreal, QC, Canada; ^4^Department of Neurology and Neurosurgery, McGill University, Montreal, QC, Canada

**Keywords:** cloud computing, reproducibility and tools, high performance computing, microservice, interactive visualization

## Abstract

Neuroscience has been carried into the domain of big data and high performance computing (HPC) on the backs of initiatives in data collection and an increasingly compute-intensive tools. While managing HPC experiments requires considerable technical acumen, platforms, and standards have been developed to ease this burden on scientists. While web-portals make resources widely accessible, data organizations such as the Brain Imaging Data Structure and tool description languages such as Boutiques provide researchers with a foothold to tackle these problems using their own datasets, pipelines, and environments. While these standards lower the barrier to adoption of HPC and cloud systems for neuroscience applications, they still require the consolidation of disparate domain-specific knowledge. We present Clowdr, a lightweight tool to launch experiments on HPC systems and clouds, record rich execution records, and enable the accessible sharing and re-launch of experimental summaries and results. Clowdr uniquely sits between web platforms and bare-metal applications for experiment management by preserving the flexibility of do-it-yourself solutions while providing a low barrier for developing, deploying and disseminating neuroscientific analysis.

## Introduction

The increasing adoption of distributed computing, including cloud and high-performance computing (HPC), has played a crucial role in the expansive growth of neuroscience. With an emphasis on big-data analytics, collecting large datasets such as the Consortium for Reliability and Reproducibility (Zuo et al., 2014), UK-Biobank (Sudlow et al., 2015), and Human Connectome Project (Van Essen et al., 2013) is becoming increasingly popular and necessary. While these datasets provide the opportunity for unprecedented insight into human brain function, their size makes non-automated analysis impractical.

At the backbone of science is the necessity that claims are reproducible. The reproducibility of findings has entered the spotlight as a key question of interest, and has been explored extensively in psychology (Open Science Collaboration, 2015), neuroimaging (Eklund et al., 2016; Bowring et al., 2018), and other domains (Baker, 2016; Miłkowski et al., 2018). Computational experiments must be re-executable as a critical condition for reproducibility, and this bare minimum requirement becomes increasingly challenging with larger datasets and more complex analyses. While sharing all code and data involved may appear a compelling solution, this is often inadequate for achieving re-runnability or reproducibility of the presented findings and models (Miłkowski et al., 2018). When re-executable applications fail to reproduce findings, there is a gray area where the source of errors are often unknown and may be linked to misinterpretation of data, computing resources or undocumented execution details, rather than scientific meaning.

As a result, new tools and standards have emerged to aid in producing more reusable datasets and tools, and thereby more reproducible science. The Brain Imaging Data Structure (BIDS) (Gorgolewski et al., 2016) and the associated BIDS apps (Gorgolewski et al., 2017) prescribe a standard for sharing and accessing datasets, and therefore, increasing the accessibility and impact of both datasets and tools. This standard includes the definition of file organization on disk, as well as key-value pairs of metadata information in JavaScript Object Notation (JSON) files, and assigns specific meaning to command-line arguments to be used when processing these datasets. The Boutiques framework (Glatard et al., 2018) provides a standard for software documentation in a machine-interpretable way, allowing the automation of tool execution and evaluation. These descriptions fully encapsulate the runtime instructions for a given tool in JSON-files, and are appropriate for a majority of command-line applications. Software containerization initiatives such as Docker (Merkel, 2014) and Singularity (Kurtzer et al., 2017) facilitate execution consistently across arbitrary computing environments with minimal burden on the user.

Web-platforms such as OpenNeuro (Poldrack et al., 2013), LONI Pipeline (Rex et al., 2003), and CBRAIN (Sherif et al., 2014) simplify the analysis process further by providing an accessible way to construct neuroscience experiments on commonly used tools and uploaded-datasets. These systems deploy tools on HPC environments and record detailed execution information so that scientists can keep accurate records and debug their workflows. Tools such as LONI’s provenance manager (Dinov et al., 2010), Reprozip (Chirigati et al., 2016), and ReCAP (Hasham et al., 2018) capture system-level properties such as system resources consumed and files accessed, where tools supporting the Neuroimaging Data Model (NIDM) (Sochat and Nichols, 2016), a neuroimaging-specific provenance model based on W3C-PROV (Missier et al., 2013), capture information about the domain-specific transformations applied to the data of interest.

The initiatives enumerated above have synergistic relationships, where each solves a small but significant piece of the larger puzzle that is computational and scientific reproducibility and replicability. However, the learning curve associated with adopting each of these technologies is considerable, leveraging them in an impactful way is difficult, and certain applications may benefit from different approaches so these learning curves may have to be paid multiple times. For instance, interoperability is mainly valuable in contexts which there is a large variety of datasets or tools, and provenance may be of importance to identify the impact of an underlying system on a processing or modeling task. We present Clowdr, a lightweight tool which ties these approaches together so that researchers can minimize the learning burden and lower the barrier to develop, perform, and disseminate reproducible, interoperable and provenance-rich neuroscience experiments.

## Emergent Technologies in Reproducible Neuroscience

Conducting reproducible analyses in neuroscience requires many complementary facets, building on technologies which are commonly adopted as *de facto* standards.

### Data and Code Interoperability

Due in part to its simplicity and active public development community, BIDS (Gorgolewski et al., 2016) has become an increasingly prominent data organization format in neuroimaging. This standard makes use of the Nifti file format (Cox et al., 2004) and human-readable JSON files to capture both imaging and subject-specific metadata. An important benefit of this data organization is the ability to launch data processing pipelines in the form of BIDS applications (Gorgolewski et al., 2017), which expose a consistent set of instructions compatible with the data organization. Together, these complementary standards are suitable for performing a large variety of neuroimaging experiments. In contexts where tools have heterogeneous interfaces, or data organizations are custom-built for a particular context, the Boutiques (Glatard et al., 2018, 2019) framework allows the rich description of a pipeline such that tool execution, validation, and exploration can be automated. These descriptors include the command-line structure to be populated as well as rich parameter descriptions and interactions, such as mutually exclusivity or dependence, such that complicated data interactions required or forbidden by the tool can be accounted for.

### Software Virtualization

While virtual machines have long been used for deploying analysis pipelines with complex dependencies in heterogeneous environments, software containers have recently emerged as lighter-weight alternatives suitable for transient data processing applications. Docker (Merkel, 2014) provides this functionality across all major host operating systems, but is often not supported by HPC centers due to security vulnerabilities (Bui, 2015; Combe et al., 2016). Singularity (Kurtzer et al., 2017) addresses the security risks of Docker, but currently only supports Linux operating systems, filling the niche of containerization on shared computing resources. A detailed comparison in the context of medical imaging can be found in Matelsky et al. (2018).

### Workflow Engines

Custom scientific pipelines can be composed in Python with Nipype (Gorgolewski et al., 2011), Dask (Rocklin, 2015), Pegasus (Deelman et al., 2015), Toil (Vivian et al., 2017), or several other tools which facilitate the modular interaction of complex independent processing stages. While the underlying tasks in Nipype, Dask, and Toil are defined in Python, Pegasus uses a Domain Specific Language (DSL) for representing tasks, increasing the barrier for defining tasks but ultimately increasing their portability. While Nipype is a widely used tool in neuroimaging and has many readily-defined interfaces available for researchers, the others require non-insignificant development to describe interfaces for common neuroimaging applications such as FSL (Jenkinson et al., 2012) or MRtrix (Tournier and Calamante, 2012). PSOM (Bellec et al., 2012) and Scipipe (Lampa et al., 2018) are functionally similar to Nipype but have been developed for GNU Octave/MATLAB and Golang, respectively. Several domains have more specialized tools which accomplish similar feats in their area of interest. These include Pypet (Meyer and Obermayer, 2016), Neuromanager (Stockton and Santamaria, 2015), Arachne (Aleksin et al., 2017), and others which facilitate the automation of modeling and simulation workflows using tools such as Neuron (Hines and Carnevale, 2001). For an in depth look at other tools in this space please refer to Meyer and Obermayer (2016) and (Stockton and Santamaria, 2015). Each of these tools enables the construction of dependency graphs between pipeline components, and allow the deployment either to cluster scheduling software, multiple processing threads, and in some cases computing clouds. These tools primarily function through programmatic interfaces, though LONI pipeline (Rex et al., 2003), OpenMOLE (Reuillon et al., 2013), and Galaxy (Goecks et al., 2010) provide both DSL and graphical user interfaces. Many of these tools also embed provenance capture, fault-tolerance features, and data tracking to avoid recomputations across similar executions. While each of these tools is a powerful and attractive option for creating workflows, they remain complex and potentially overkill when launching atomic single-step analyses, prebuilt pipelines, or analysis software developed in a different language than the workflow engine of choice.

### Provenance

Building on the W3-PROV (Missier et al., 2013) standard for data provenance metadata put forth by the World Wide Web Consortium, NIDM (Sochat and Nichols, 2016) is a standard which represents a processing and data provenance graph specific to neuroimaging analyses. While this standard is machine-interpretable and interoperable-by-design, supporting it currently requires tight integration with analysis pipelines. In LONI pipeline, a provenance model exists which includes detailed records of data use and file lifecycle (Dinov et al., 2010), which is designed to inform data consumers what types of analyses can be and have been performed with the data in question; this tool is tightly coupled with the LONI pipeline ecosystem. The ReCAP (Hasham et al., 2018) project has been developed to evaluate the resource consumption of arbitrary pipelines on the cloud and can aid in cloud-instance optimization. While this tool has potential for a large impact in designing both cost effective and scalable analyses, there is considerable overhead as it manages executions through a persistent server and workflow engine. While various other libraries exist to monitor some piece of data or compute provenance, Reprozip (Chirigati et al., 2016) is perhaps the most exciting as it uniquely captures records of all files accessed and created throughout an execution, which allows for the creation of rich file dependency graphs. The limitation of this technique is that it requires data of interest to be written to disk, as opposed to managed in memory, which may not always be the case in some applications.

### Web Platforms

Increasing the portability and accessibility of launching large scale analyses, web platforms such as CBRAIN (Sherif et al., 2014), LONI pipeline (Rex et al., 2003), and OpenNeuro (Poldrack et al., 2013) provide science-as-a-service where users can upload and process their data on distant computing resources. Additionally, these platforms provide an accessible and immediate way to share the results produced from experiments with collaborators or the public. These tools provide incredible value to the community and allow the deployment of production-level pipelines from the web, but they are not suitable for prototyping analyses or developing pipelines, and it is cumbersome to run these services on a lab’s own resources. In addition, monolithic Web interfaces are only suitable for a certain type of use-cases and high-level users, while developers or computer-savvy users prefer to rely on modular command-line tools and libraries.

## The Clowdr Microtool

While the technologies enumerated above are essential pieces toward reproducible neuroscience, they are largely isolated from one another and place a large burden on researchers who wish to adopt all of these best practices. Clowdr leverages these tools to increase the deployability, provenance capture, and shareability of experiments. In summary, Clowdr:

(i) is tightly based on Boutiques and is BIDS-aware, supporting both arbitrary pipelines and providing an accessible entrypoint for neuroimaging;
(ii) executes both bare-metal workflows and Docker or Singularity virtualized pipelines through Boutiques on local, HPC, and cloud resources;
(iii) supports the parallelized batch deployment and redeployment of pipelines constructed with workflow-engines, while being agnostic to programming language and construct;
(iv) captures system-level provenance information (i.e., CPU and RAM usage), supports Reprozip, and internal provenance captured by arbitrary pipelines such as NIDM; and
(v) supports the deployment of both development- and production-level tools without an active server, and provides a web-report for exploring and sharing experiments.

**FIGURE 1:**
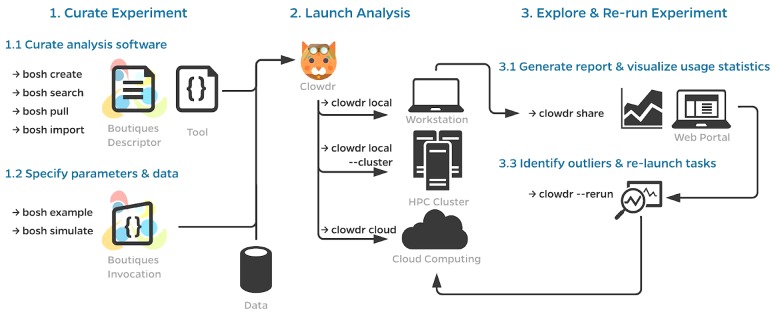
Clowdr Workflow. **(1)** Prior to launching an analysis with Clowdr, users must curate the analysis tools and their inputs. For the sake of portability, Clowdr supports both native and containerized applications described in the Boutiques format. Several tools exist in Boutiques which simplify the adoption/creation or execution of tools and are enumerated in **(1.1,1.2)**, respectively. **(2)** Scientists can then launch their analysis with Clowdr either locally, on HPC systems, or computing clouds. Possible workflows could involve the tuning of hyperparameters locally on a subset of the dataset of interest, and ultimately deploying the analysis at scale using the same arguments, or sweeping hyperparameter values on an HPC system. **(3)** After execution, summary reports can be produced by Clowdr **(3.1)** and visualized through a custom web portal enabling filtering by both execution properties and parameters, facilitating outlier detection and comparison across executions. Identified outliers, such as failures, incomplete tasks, or those which consumed more resources than expected can be re-run through Clowdr without having to regenerate any of the information previously provided. Clowdr facilitates the development, deployment, and debugging of analyses in a closed-loop provenance-rich microservice.

A typical workflow using Clowdr is summarized in Figure 1. While a Clowdr experiment follows the same workflow as traditional experiments, beginning with tool and data curation through prototyping, deployment, and exploration, there are several considerable benefits provided by Clowdr over traditional approaches. In particular, Clowdr is based on the rich Boutiques framework for tool description and execution, ensuring that documentation, parameter definitions, and real-world parameter values accompany the tool at all times. Clowdr also treats all computing systems the same, from the users perspective, so transitioning from local development of analyses to at-scale systems is seamless, which minimizes errors made during this transition. Clowdr also provides a visualization portal for exploring executions and filtering either based on parameter values or runtime statistics, allowing for quality control of the execution in addition to commonly used quality control of processed derivatives themselves.

**FIGURE 2:**
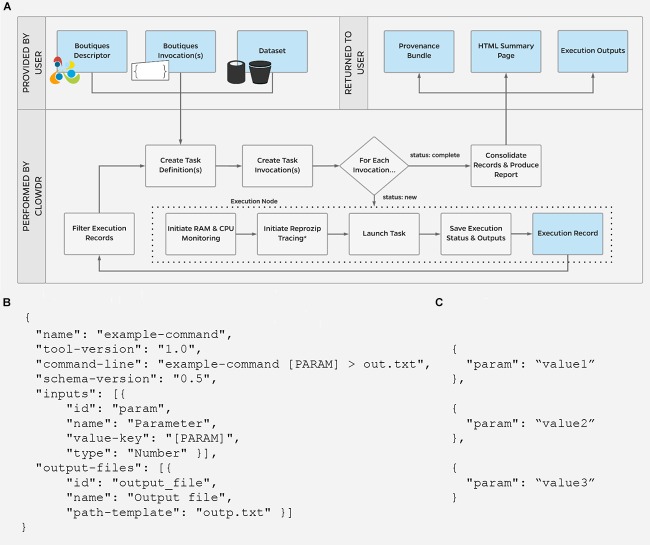
**(A)** Clowdr Data Flow. Beginning with a user-supplied tool descriptor **(B)** and parameter invocation(s) **(C)**, Clowdr identifies unique tasks to launch and wraps each with usage and log monitoring tools, to ultimately provide a rich record of execution to the user alongside the expected output products of the experiment. Clowdr ultimately produces an HTML summary for users to explore, update, filter, and share the record of their experiment. In the above schematic, blue boxes indicate data, where gray indicate processing steps. ^∗^External reprozip tracing is supported on limited infrastructures, as running virtualized environments within a trace capture requires elevated privileges which may be a security risk on some systems.

Figure 2 shows the execution lifecycle within Clowdr. Starting from user-provided Boutiques descriptor (B) and invocation(s) (C), and access to any required datasets, Clowdr begins by identifying a list of tasks to launch. For a new experiment, tasks are identified in one of three main ways: (1) a one:one mapping from a list of invocations, (2) a one:many mapping from a single invocation in which parameter(s) have been specified for sweeping during execution, or a BIDS-specific, and (3) one:many mapping from a single invocation for a BIDS app, which will iterate upon the participant- and session-label fields, and described in the BIDS app specification (Gorgolewski et al., 2017). Experiments can be re-run, and determine the task-list based on whether a full, failure-only, or incomplete-only re-execution is desired. Once the task-list is determined, Clowdr creates an independent invocation which explicitly defines the arguments used in each task.

At this stage, Clowdr distributes tasks to the Cloud system or local cluster scheduler being used for deployment. Presently Clowdr supports the SLURM scheduler and Amazon Web Services (AWS) cloud through their Batch service with adoption of more platforms ongoing. Each task is launched through a Clowdr-wrapper, which initializes CPU and RAM monitoring and triggers Reprozip tracing prior to launching the analysis itself. Reprozip tracing has limited support in conjunction with containerized analyses on HPC systems due to potential security issues. Reprozip is built upon the Linux command “ptrace,” which traces processes to monitor or control them. To eliminate the potential risk of using this tool, it is common for systems to disallow the tracing of administrator-level processes. The requirement of limited administrator privileges by Singularity (during the creation of multiple user namespaces) and Docker (for interacting with the daemon) makes encapsulating these tools within the restricted ptrace scope not possible on many shared systems. For more information on the specific conditions in which these technologies can be made to interoperate please view the GitHub repository for this manuscript, linked below.

Upon completion of the analysis, Clowdr bundles the system monitored records, standard output and error, exit status, and any other information collected by either the tool itself or the Boutiques runtime engine, and concludes its execution. Once the experiment has begun, Clowdr provides the user with the Clowdr provenance directory which will be updated automatically as executions progress.

**FIGURE 3:**
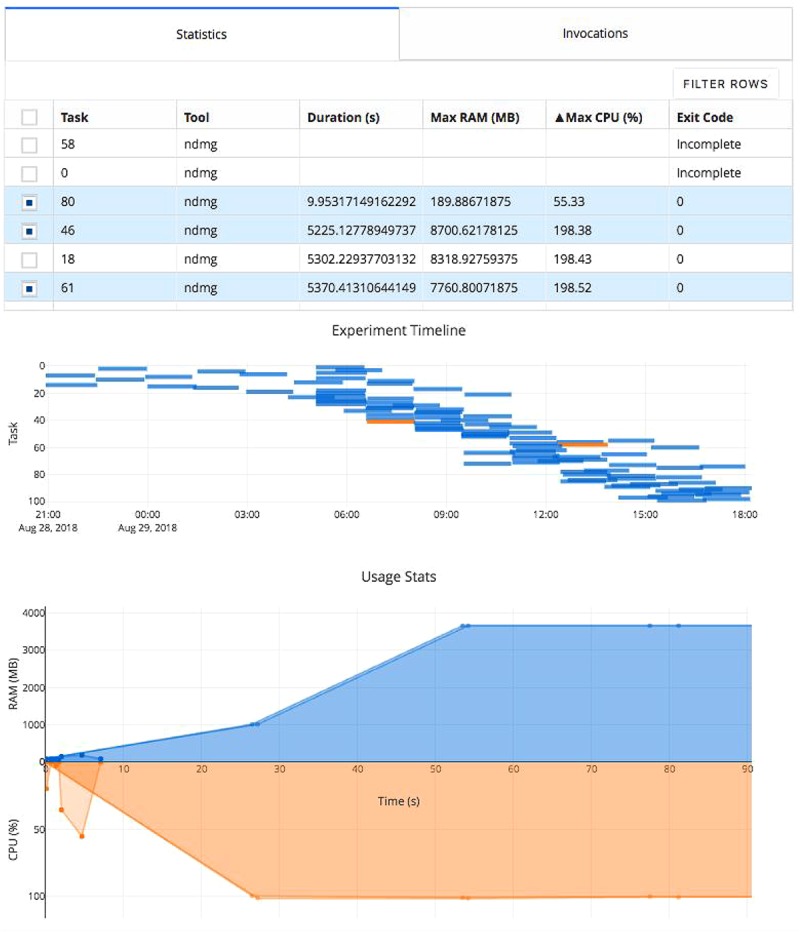
Clowdr Experiment Viewer. Experiments launched with Clowdr can be monitored and both progress and runtime statistics explored. The page is produced using Plotly Dash to produce highly interactive plots and tables, enabling rich filtering, rescaling, and exploration of executions. The table can be toggled to present summary statistics about experiment execution or invocation parameters identifying parameters used for each task in the experiment. The subsequent Gantt plot shows the timeline of executed tasks in the experiment, where those selected for visualization in the usage plot below are highlighted. The final plot in this view shows the memory and processing footprint throughout all selected tasks. Selection and filtering may be done by value in the tables or selection in the task timeline. In this example, several tasks did not complete and one appeared to exit after 10 s erroneously. The Clowdr portal enables quick identification of these outliers, and the table view can be switched to identify more information such as parameters pertaining to the executions of interest. For more information about the pipeline being executed in particular, please see Kiar et al. (2018b).

The researcher can monitor the provenance directory using the Clowdr share portal (Figure 3), which provides a web interface summarizing the task executions. Once the analysis concludes, the figures on this web page and the associated metadata can be saved and serve as a record of the experiment either for evaluation or dissemination alongside published results.

The Clowdr package is open-source on GitHub (Kiar and Glatard, 2019), and installable through the Python Package Index.

## Performing Experiments With Clowdr

Here, we explore an experiment in which we used Clowdr to process the Human Connectome Project (HCP) (Van Essen et al., 2013) dataset with a structural and functional connectome estimation pipeline, ndmg (Kiar et al., 2018a,b). The records of this experiment, and materials and instructions that can be used to reproduce a similar analysis with Clowdr using the publicly available DS114 BIDS dataset (Poldrack et al., 2013) an the example BIDS application (Gorgolewski et al., 2017) can be found on Github at: https://github.com/clowdr/clowdr-paper. Specific packages and their versions for both experiments can be found at the end of this manuscript.

As summarized above, performing an analysis with Clowdr requires the creation of a Boutiques descriptor summarizing the pipeline of interest, an invocation containing the parameters to supply to this pipeline on execution, and curation of the data to be processed. There are several utilities in Boutiques which aid in this setup process, including to automatically generate a descriptor and sample inputs from a tool, and can be explored in the associated documentation (Glatard et al., 2019).

Clowdr experiments can be launched locally, on cluster, or submitted to cloud resources. In each case, invocations and task definitions are created locally, and then the jobs are run serially, submitted to a cluster queue, or pushed to cloud storage and called remotely. The commands used in Clowdr to launch these commands are the local, local with the cluster switch, and cloud modes, respectively. Upon completion of each tasks, summary files created by Clowdr can be either inspected manually or consolidated and visualized in the web with the Clowdr share command (Figure 3).

The share tool, launchable on any computer with access to the experiment, creates a lightweight web service displaying summary statistics and invocation information from the experiment, including memory usage, task duration, launch order, and log information. The visualizations provided are filterable and sortable, enabling users to interrogate and identify outliers in their experiment, explore potential sources of failure, and effectively profile the analysis pipeline in use. The modified figures can be downloaded from this interface, serving as accessible records of execution.

In the example above, the HCP dataset has been processed using a pipeline performing image denoising, registration, model fitting, and connectivity estimation, all of which are commonly used processing steps in neuroimaging. For more information on this pipeline, please see Kiar et al. (2018b).

In this experiment the table has been filtered to show several tasks which appeared spurious in their execution compared to the others. We can see that several tasks failed to complete and one appeared to terminate in significantly less time than the others. After identifying these tasks and exploring the time series’ to see at what stage of processing the job failed (in this case, immediately), we can investigate parameter selections used in each and attempt the re-execution of these jobs using the local or cloud command with the rerun switch in Clowdr. Clowdr provides a layer of quality control on executions, in addition to that which is regularly performed by researchers on their datasets, which provides immediate value when identifying task failures which otherwise may be difficult to identify, especially in cases which intermediate and terminal derivatives are written to the same location, which can often be the case with transformations estimated by registration pipelines, for example.

While the share tool currently requires maintaining an active server, the plots can be exported statically and it is in the development roadmap to enable exporting the entire web page as static files, as discussed here: https://github.com/plotly/dash/issues/266. Since the record created by Clowdr is stored in the machine-readable and JSON format, researchers can easily extract their records and integrate it into other interfaces that suit their application.

## Discussion

Clowdr addresses several barriers to performing reproducible neuroscience. Clowdr experiments consist of enclosed computational environments, versioned-controlled Boutiques-described tools with explicit usage parameters, rich execution history, and can be re-executed or distributed with minimal effort. Clowdr provides an accessible interface for initially running analyses locally, and translating them seamlessly to HPC environments. The rich record keeping provided with Clowdr is system-agnostic resulting in uniformly interpretable summaries of execution. As a Python library, Clowdr can be used as a module in a larger platform, or directly as a command-line tool.

Clowdr uniquely packages an executable tool summary, parameters, and results together, in a language- and tool-agnostic way, and therefore, greatly increases the transparency and shareability of experiments. Importantly, this adds clarity to experimental failures and documents the hyper-parameter tuning process of experiments, which has been historically largely undocumented in literature (Reunanen, 2003).

There are several axes upon which the value of Clowdr can be discussed. In particular: lines of code written, time spent, and the ultimate re-runnability of analyses. While these remain subjective areas for comparison, we can conceptually consider a workflow dependent on Clowdr to those constructed with traditional scripting, workflow engines (WEs), and software-as-a-service (SaaS) platforms.

Where Clowdr has been built upon tools and standards to provide users with a series of single-commands for launching and managing analyses, accomplishing a similar result with traditional scripting would take considerably more lines of code and time. Similarly, where command-line execution may be similar in complexity to tools developed with WEs, their integration within tools requires substantial development and is only practical in cases for which there is a WE written in the same language as the underlying application. SaaS platforms provide a similar type of abstraction to Clowdr, where tools are treated as black-box objects, but come with the added overhead of maintaining complex database architectures, often complex integration of tools, and primarily restrict access through web-based interfaces which leads to reduced flexibility for the user.

The clear benefit of Clowdr is in the simplicity it provides for identifying outliers or failed tasks and either re-launching specific subsets of an analysis or the entire experiment. Clowdr records and visualizes detailed logging information about executions and the specific instructions which were used, which isn’t guaranteed in either traditional scripting or WE-based applications. To replicate this feature across these systems, tools which (1) record execution instructions, (2) identify parameters used for parallelization, (3) produce summary plots, and (4) reconstruct and (5) re-execute instructions would require development.

While SaaS platforms often contain these features, an additional limitation of large platforms is that they are often designed for consumers of widely adopted tools consumers rather than tool developers. Clowdr fills the void between these types of pipeline deployments by providing a programmatic tool-independent method for managing job submission and collecting provenance across multiple architectures and enabling the rapid prototyping of analyses.

Several immediate applications of the provenance information captured by Clowdr include the benchmarking of tools, and resource optimization during the selection of cloud resources, as was done in Hasham et al. (2018). While the value of comparing provenance records has not been demonstrated here, other studies such as (Salari et al., 2018) have demonstrated the efficacy of leveraging provenance information to identify sources of variability or instability within pipelines.

Future work includes adopting a W3C-PROV compatible format for Clowdr provenance records, increasing the machine-readability and interoperability of these records with other standards such as NIDM. Integrating the reports produced by Clowdr with a system such as Datalad would allow for record versioning and more strictly enforce the complete reporting of experiments. Clowdr will also continually be extended with greater testing and support for more HPC schedulers, clouds, and provenance capture models.

## Tools and Versions

The following is a list of tools and data used in this manuscript, and their respective versions. The architecture and analysis presented for the Clowdr package corresponds to version 0.1.0. The key Python packages and specific versions tested are: boutiques (version 0.5.12), boto3 (1.7.81), botocore (1.10.81), slurmpy (0.0.7), psutil (5.4.7), pandas (0.23.4), plotly (3.1.1), and plotly dash (0.24.1), including dash-core-components (0.27.1), dash-html-components (0.11.0), dash-renderer (0.13.0), dash-table-experiments (0.6.0), and flask (0.12.2). Executions were tested locally using Docker (17.12.0-ce), and on Compute Canada’s Cedar high performance cluster using Singularity (2.5.1-dist). The Docker container used for ndmg can be found on Docker hub as neurodata/m3r-release (0.0.5), which contains ndmg (0.1.0-f). The Singularity container used was pulled and dynamically created from this Docker hub endpoint. The dataset use was a subset of the HCP 1200 collection (Van Essen et al., 2013).

## Author Contributions

GK designed and developed the tools, experiments, and figures, and wrote the majority of the manuscript. SB supported the design and development processes, and edited the manuscript and provided valuable feedback. TG provided insight and contributed to the design and development of the tools and experiments, and contributed to writing the manuscript. AE edited the manuscript and provided valuable feedback. TG and AE jointly supervised this project.

## Conflict of Interest Statement

The authors declare that the research was conducted in the absence of any commercial or financial relationships that could be construed as a potential conflict of interest.
